# β-TrCP- and Casein Kinase II-Mediated Degradation of Cyclin F Controls Timely Mitotic Progression

**DOI:** 10.1016/j.celrep.2018.08.076

**Published:** 2018-09-25

**Authors:** Ioanna Mavrommati, Roberta Faedda, Giovanni Galasso, Jie Li, Kamila Burdova, Roman Fischer, Benedikt M. Kessler, Zunamys I. Carrero, Daniele Guardavaccaro, Michele Pagano, Vincenzo D’Angiolella

**Affiliations:** 1 Cancer Research UK and Medical Research Council Institute for Radiation Oncology, Department of Oncology, University of Oxford, Old Road Campus Research Building, Roosevelt Drive, Oxford OX3 7DQ, UK; 2 Department of Biochemistry and Molecular Pharmacology, New York University School of Medicine, 522 First Avenue, SRB 1107, New York, NY 10016, USA; 3 NYU Perlmutter Cancer Center, New York University School of Medicine, 522 First Avenue, SRB 1107, New York, NY 10016, USA; 4 Howard Hughes Medical Institute, New York University School of Medicine, 522 First Avenue, SRB 1107, New York, NY 10016, USA; 5 Target Discovery Institute, Nuffield Department of Medicine, University of Oxford, Old Road Campus, Roosevelt Drive, Oxford OX3 7FZ, UK; 6 Hubrecht Institute-KNAW and University Medical Center Utrecht, Uppsalalaan 8, 3584 CT Utrecht, the Netherlands; 7 Department of Biotechnology, University of Verona, Strada Le Grazie 15, 37134 Verona, Italy

**Keywords:** F-box protein, cyclin F, β-TrCP, mitosis, cell cycle, ubiquitin, SCF, CRLs, CKII

## Abstract

Orderly progressions of events in the cell division cycle are necessary to ensure the replication of DNA and cell division. Checkpoint systems allow the accurate execution of each cell-cycle phase. The precise regulation of the levels of cyclin proteins is fundamental to coordinate cell division with checkpoints, avoiding genome instability. Cyclin F has important functions in regulating the cell cycle during the G2 checkpoint; however, the mechanisms underlying the regulation of cyclin F are poorly understood. Here, we observe that cyclin F is regulated by proteolysis through β-TrCP. β-TrCP recognizes cyclin F through a non-canonical degron site (TSGXXS) after its phosphorylation by casein kinase II. The degradation of cyclin F mediated by β-TrCP occurs at the G2/M transition. This event is required to promote mitotic progression and favors the activation of a transcriptional program required for mitosis.

## Highlights

• β-TrCP1 and β-TrCP2 interact with cyclin F and control cyclin F levels during mitosis
• A TSGXXS motif is necessary for β-TrCP1 and β-TrCP2 binding to cyclin F
• CKIIα phosphorylates cyclin F at S704 within the TSGXXS motif
• β-TrCP-mediated degradation of cyclin F promotes mitotic progression via B-Myb

## Graphical Abstract

**Figure:**
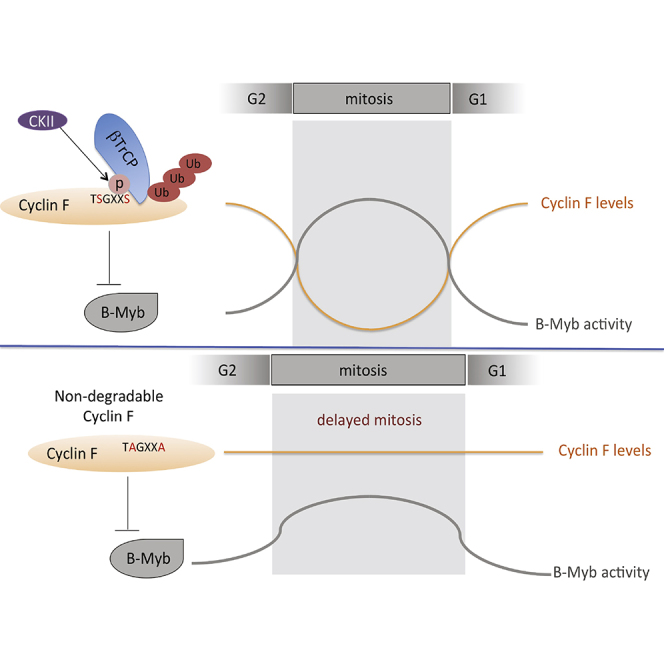

## Teaser

Mavrommati et al. identify how cyclin F is regulated during mitosis by proteolysis. Cyclin F is ubiquitylated by β-TrCP after phosphorylation by CKII on S704 within a TSGXXS degron. β-TrCP-mediated degradation of cyclin F favors timely mitotic progression through the control of a B-Myb transcriptional program.

## Introduction

Cell-cycle transitions are operated by the periodic oscillations of cyclins, which bind cyclin-dependent kinases (CDKs) to phosphorylate target substrates. Among the cyclins coordinating cell-cycle progression, cyclin F’s amino acid sequence is most similar to cyclin A2, but cyclin F does not act as an activator of CDKs. Instead, cyclin F (Fbxo1) is the founding member of the F-box family of proteins (Bai et al., 1996). These 69 proteins share an F-box domain, necessary for binding to Skp1. Skp1 recruits Cul1, which in turn binds Rbx1, a protein required for interaction with the UBC3 E2 enzyme. Cyclin F, using the F-box domain, forms a functional Skp1-Cul1-F-Box (SCF) complex, mediating the ubiquitylation and degradation of target proteins (D’Angiolella et al., 2013). Among the cyclin F substrates identified so far are CP110, a centrosomal protein important for centrosome duplication (D’Angiolella et al., 2010); NUSAP, a nucleolar and spindle-associated protein (Emanuele et al., 2011); RRM2, a subunit of ribonucleotide reductase (D’Angiolella et al., 2012); Exo1, an exonuclease required for genome stability (Elia et al., 2015); Cdc6, an essential component of DNA replication origin selection (Walter et al., 2016); and SLBP, a stem-loop binding protein (Dankert et al., 2016). In addition to regulating substrates through proteolysis, cyclin F can counteract the activity of cyclin A2 by competing with cyclin A2 substrates’ recognition. It has been shown that cyclin F interacts with B-Myb to prevent the access of cyclin A2 and the consequent hyper-phosphorylation of B-Myb by cyclin A2. By this means, cyclin F is acting to control the transcriptional program required for mitosis (Klein et al., 2015). A common feature of the substrates and interacting partners in which the recognition motif has been identified is that cyclin F recognizes an RxL motif in the substrates through a hydrophobic patch in the cyclin domain. Thus, the recognition mechanism that cyclin F uses to engage substrates is the same as for any other cyclin, with the major difference being that cyclin F has an inhibitory function. This is mainly exerted through degradation of downstream substrates.

Cyclin F’s levels oscillate throughout the cell cycle similar to canonical cyclins. Protein levels of cyclin F are low during mitosis and G1, increase in S, and peak in the G2 phase of the cell cycle (D’Angiolella et al., 2012, Fung et al., 2002). Cyclin F must be tightly regulated during cell-cycle progression to avoid unscheduled degradation of cyclin substrates. Therefore, we investigated the mechanisms of cyclin F regulation during the cell cycle and identified β-TrCP as an E3 ubiquitin ligase interacting with cyclin F. β-TrCP is also an F-box protein, and it uses its WD40 domains to recruit substrates that are phosphorylated within a degron sequence (DSGXXS) (Guardavaccaro and Pagano, 2006, Skaar et al., 2013). Although the regulation of F-box protein stability is largely ascribed to autoubiquitylation, the possibility that F-box proteins might regulate each other has been postulated based on both mechanistic studies (Guardavaccaro et al., 2003) and large-scale screenings (Emanuele et al., 2011). However, to date, only a few examples of this interplay have been described. Here, we show that cyclin F is targeted for degradation by β-TrCP during the G2/M transition of the cell division cycle. The degradation of cyclin F via β-TrCP is initiated by phosphorylation of cyclin F by casein kinase IIα (CKIIα). The proteolysis of cyclin F is necessary to ensure timely mitotic progression.

## Results

### β-TrCP1 and β-TrCP2 Interact with Cyclin F and Control Cyclin F Protein Levels during Mitosis

**Figure 1:**
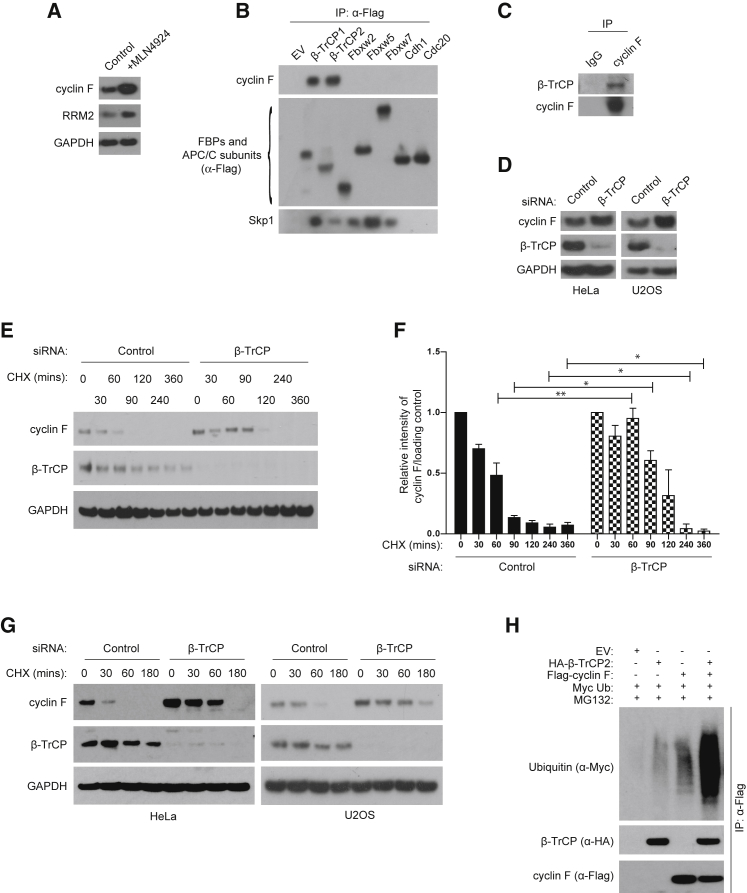
β-TrCP1 and β-TrCP2 Interact with Cyclin F and Control Cyclin F Protein Levels during Mitosis (A) HEK293T cells treated with MLN4924 (2 μM) for 4 hr were immunoblotted as indicated. GAPDH was the loading control. (B) HEK293T cells transfected with an empty vector (EV), indicated FLAG-tagged F-box proteins (FBPs), CDH1, or Cdc20 were immunoprecipitated (IP) with anti-FLAG resin, and immunoblotted as indicated. (C) Endogenous cyclin F was IP from HEK293T cell extracts using cyclin F antibody or non-specific rabbit immunoglobulin (IgG) as a loading control and immunoblotted as indicated. (D) HeLa cells (left panel) and cells U2OS (right panel) transfected with a non-targeting siRNA (control) or β-TrCP1/2 siRNA for 48 hr were immunoblotted as indicated. (E) HeLa cells transfected as in (D) were treated with cycloheximide (CHX) for the indicated minutes and immunoblotted as indicated. (F) Western blot densitometry analysis of cyclin F. Results are means ± SEM of three independent experiments. Student’s t test. ^∗^p < 0.05, ^∗∗^p < 0.01. (G) HeLa cells (left panel) and U2OS cells (right panel) transfected as in (D) were synchronized by a thymidine-nocodazole block. Cells were then treated with cycloheximide (CHX) for the indicated minutes and immunoblotted as indicated. (H) HEK293T cells transfected with the indicated plasmids were treated with MG132 (10 μM), immunoprecipitated (IP) with anti-FLAG resin, and immunoblotted as indicated.

The anaphase-promoting complex/cyclosome (APC/C) has been shown to mediate the degradation of cyclin F in G1 (Choudhury et al., 2016), but the degradation of cyclin F in mitosis is controlled by an unknown mechanism. We found that levels of cyclin F are drastically increased by treatment of cells with MLN4924 (Figure 1A), an inhibitor of NAE (Nedd8-activating enzyme) (Soucy et al., 2009). Because the activity of NAE is required for the function of SCF ligases, we tested the hypothesis that cyclin F could be regulated by either an autocatalytic mechanism or a different F-box protein. Thus, we screened a panel of FLAG-tagged F-box proteins and substrate receptors of the APC/C expressed in HEK293T cells for the binding to cyclin F. All F-box proteins interacted with Skp1, but β-TrCP1 and β-TrCP2 were the only two E3 ligases able to bind to endogenous cyclin F (Figure 1B). The interaction between endogenous cyclin F and endogenous β-TrCP after immunoprecipitation of cyclin F was detected (Figure 1C).

To assess whether β-TrCP affects cyclin F protein levels, we measured cyclin F protein levels upon downregulation of β-TrCP1/2 using small interfering RNA (siRNA). Compared to non-targeted control cells, β-TrCP1/2 downregulation increased cyclin F protein levels in both HeLa and U2OS cells (Figure 1D), suggesting that β-TrCP1 and β-TrCP2 control cyclin F stability. We also measured the half-life of cyclin F after siRNA of β-TrCP1/2. Although cyclin F half-life was 60 min in non-targeted control cells, the half-life was prolonged to 90 min upon β-TrCP1/2 silencing (Figure 1E). This effect was quantified by densitometry analysis of three independent experiments (Figure 1F).

To test whether cyclin F is targeted for degradation by β-TrCP1/2 in a specific cell-cycle phase, we synchronized HeLa cells using a double thymidine block (DTB) and silenced β-TrCP1/2. Compared to control cells, downregulation of β-TrCP1/2 during mitosis resulted in increased levels of cyclin F, as well as reduced histone H3 phosphorylation at serine 10 and prolonged CDC2 phosphorylation at threonine 14 and tyrosine 15 (Figure S1A). This effect of delayed progression in mitosis, mediated by β-TrCP1/2 siRNA, is likely due to the accumulation of Wee1, a substrate of β-TrCP1/2 that prevents mitotic entry (Watanabe et al., 2004). To avoid the delayed mitotic entry induced by β-TrCP1/2 siRNA, we performed siRNA of β-TrCP1/2 and measured the half-life of cyclin F in cells arrested in mitosis after nocodazole treatment. The half-life of cyclin F upon siRNA of β-TrCP1/2 in HeLa and U2OS mitotic arrested cells was increased to 60 min, compared to 30 min in non-targeted control cells (Figure 1G). The increased half-life was quantified by densitometry analysis in three independent experiments (Figures S1B and S1C). Finally, we tested whether cyclin F can be ubiquitylated by β-TrCP2. Expression of β-TrCP2 drastically increased cyclin F ubiquitylation (Figure 1H).

These results show that β-TrCP mediates the ubiquitylation and degradation of cyclin F to control its protein levels in mitosis.

### A Non-canonical DSGXXS Motif Is Necessary for β-TrCP Binding to Cyclin F

**Figure 2:**
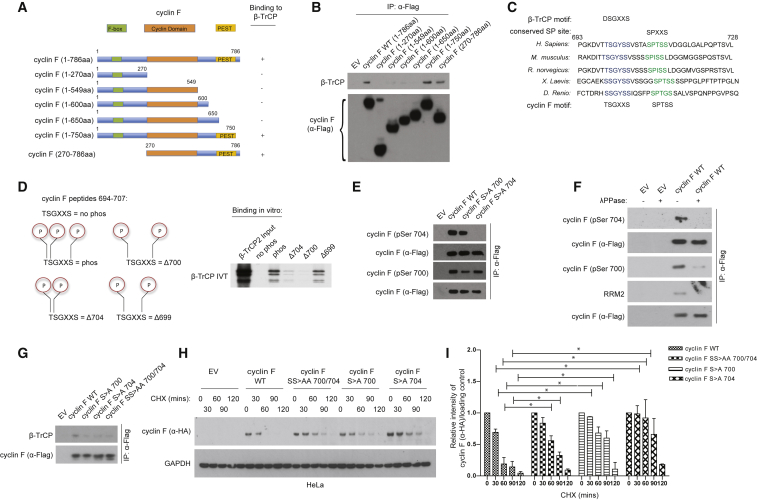
A Non-canonical DSGXXS Motif Is Necessary for β-TrCP1 and β-TrCP2 Binding to Cyclin F (A) Schematic representation of cyclin F WT and truncated fragments, highlighting F-box, the cyclin domain, and PEST. Cyclin F fragments interacting with endogenous β-TrCP are designated with the + symbol. (B) HEK293T cells transfected with an empty vector (EV), FLAG-tagged cyclin F WT, or FLAG-tagged cyclin F truncated fragments were immunoprecipitated (IP) and immunoblotted as indicated. (C) Alignment of cyclin F orthologs highlighting the putative β-TrCP binding motif and a conserved serine proline (SP) site. (D) *In vitro*-transcribed and -translated β-TrCP2 labeled with methionine ^35^S was incubated at 30°C with beads coupled to the following peptides: 694-GKDVTTSGYSSVST-707 (no-phos), 694-GKDVTTpSpGYSSpVST-707 (phos), 694-GKDVTTSpGYSSpVST-707 (Δ699), 694-GKDVTTpSGYSSpVST-707 (Δ700), and 694-GKDVTTpSpGYSSVST-707 (Δ704). After 30 min, beads were washed, and bound β-TrCP2 was detected by autoradiography. (E) HEK293T cells transfected with indicated plasmids were IP with anti-FLAG and immunoblotted as indicated. (F) HeLa cells stably expressing an empty vector (EV) and FLAG-tagged cyclin F WT were IP. Immunoprecipitates were dephosphorylated by treatment with lambda phosphatase (λPPase±) and immunoblotted as indicated. (G) HeLa cells stably expressing cyclin F WT and S > A 700, S > A 704, and SS > AA 700/704 mutants were IP and immunoblotted as indicated. (H) HeLa cells stably expressing cyclin F WT and S > A 700, S > A 704, and SS > AA 700/704 were treated with cycloheximide (CHX) in minutes and immunoblotted as indicated. (I) Densitometry analysis of cyclin F WT and S > A 700, S > A 704, and SS > AA 700/704 half-life. Results are means ± SEM of three independent experiments. Student’s t test. ^∗^p < 0.05.

β-TrCP1 and β-TrCP2 recruit their substrates using their WD40 domains, which interact with two phosphorylated serine residues in a DSGXXS sequence (Kim et al., 2014). Cyclin F does not contain a β-TrCP canonical recognition sequence; therefore, we searched for the β-TrCP binding region on cyclin F using cyclin F truncation mutants (Figure 2A). β-TrCP binds to full-length, wild-type (WT) cyclin F and two truncated forms of cyclin F encompassing amino acids (aa) 650 to 750 (Figure 2B). We found that the β-TrCP binding domain in cyclin F is located outside the cyclin domain and within a PEST sequence (rich in proline, glutamic acid, serine, and threonine).

Since β-TrCP1 and β-TrCP2 are highly conserved along evolution, we reasoned that the regulation of cyclin F by β-TrCP would be also a conserved feature across species. Therefore, we aligned the region of human cyclin F (aa 650–750) containing the binding site for β-TrCP to the amino acid sequence of cyclin F from other species. A highly conserved region within the cyclin F sequence contains T/SSGXXS, which closely resembles the canonical DSGXXS motif of other β-TrCP substrates (Figure 2C). The alignment analysis also revealed a highly conserved residue at position S709 (Figure 2C).

Next, we asked whether the TSGXXS residues are sufficient for β-TrCP binding. To this end, we generated peptides from aa 694 to 707 containing the TSGXXS motif, where T699, S700, and S704 are differentially phosphorylated. Specifically, we generated a non-phosphorylated (no-phos) peptide; a peptide for which T699, S700, and S704 are phosphorylated (phos); a peptide for which only S699 and S700 are phosphorylated (Δ704); a peptide for which only T699 and S704 are phosphorylated (Δ700); and a peptide for which only S700 and S704 are phosphorylated (ΔT699). We tested the *in vitro* binding of these peptides to β-TrCP2 after transcription-translation of β-TrCP2 using an *in vitro* transcription and translation (IVT) reticulocyte system. Using this approach, we observed that the phosphorylation of residues 700 and 704 within TSGXXS is necessary and sufficient for binding to β-TrCP2, whereas the phosphorylation of T699 affects the interaction with β-TrCP2 of only approximately 30% (Figure 2D).

To address whether the phosphorylation of cyclin F occurs *in vivo* and is relevant for β-TrCP binding, we developed phospho-specific antibodies detecting phosphorylated S700 and S704. The antibody raised against S704 detected WT cyclin F, but not a cyclin F mutant for which the S704 residue was changed to alanine (S > A 704), indicating that cyclin F is phosphorylated *in vivo* and the antibody is specific (Figure 2E). The antibody detecting S700 recognized cyclin F WT and a weaker band when cyclin F S700 was mutated to alanine (S > A 700), suggesting that the antibody is recognizing phosphorylated cyclin F on S700, even though it recognizes non-phosphorylated cyclin F with lower affinity. The S700 and S704 residues are phosphorylated independently of each other, because we detected S704 phosphorylation when S700 was mutated to alanine, and vice versa (Figure 2E).

To ensure that the specificity of recognition of the antibodies was due to a lack of phosphorylation and not the change in the amino acid residues in cyclin F, we immunoprecipitated cyclin F and dephosphorylated it using a non-specific phosphatase (λ). As a positive control of the dephosphorylation event, we tested the interaction between cyclin F and RRM2. We have previously shown that the interaction between cyclin F and RRM2 depends on the phosphorylation of RRM2 on T33, which unmasks the degron recognized by cyclin F (D’Angiolella et al., 2012). After dephosphorylation of cyclin F immunoprecipitates, we observed loss of interaction between cyclin F and RRM2 and loss of recognition of cyclin F using the anti-phospho-S704 antibody (Figure 2F). Using the anti-phospho-S700 antibody, we observed a reduction, but not the elimination, of the signal, confirming that this antibody can also recognize non-phosphorylated cyclin F with low affinity.

The prediction from the preceding experiments is that the lack of phosphorylation at residues S700 and S704 impairs the binding of cyclin F to β-TrCP. To this end, we generated HeLa cell lines expressing either cyclin F WT or cyclin F S > A 700, S > A 704, or SS > AA 700/704 mutants. The expression of cyclin F WT and S > A 700, S > A 704, or SS > AA 700/704 mutants was similar in all cell lines, and it was lower than the levels of endogenous cyclin F (Figure S2A). Compared to cyclin F WT, the binding of cyclin F S > A 700, S > A 704, or SS > AA 700/704 mutants to β-TrCP was compromised (Figure 2G). We then measured the half-life of cyclin F WT and cyclin F mutants. Although the half-life of cyclin F WT in HeLa cells was ∼30 min, the half-life of cyclin F mutants was more than 90 min (Figure 2H). The effect was quantified by densitometry analysis of three independent experiments (Figure 2I). We also tested the half-life of cyclin F WT, S > A 700, S > A 704, and SS > AA 700/704 mutants in U2OS. We found that the half-life cyclin F mutants lacking critical residues for β-TrCP recognition was increased (Figure S2B, quantified in Figure S2C), indicating that the regulation of cyclin F by β-TrCP represents a general cell-cycle control mechanism.

Overall, the data demonstrate that β-TrCP initiates degradation of cyclin F after recognition of a TSGXXS motif in which the residues S700 and S704 need to be phosphorylated.

### CKIIα Phosphorylates Cyclin F at S704

**Figure 3:**
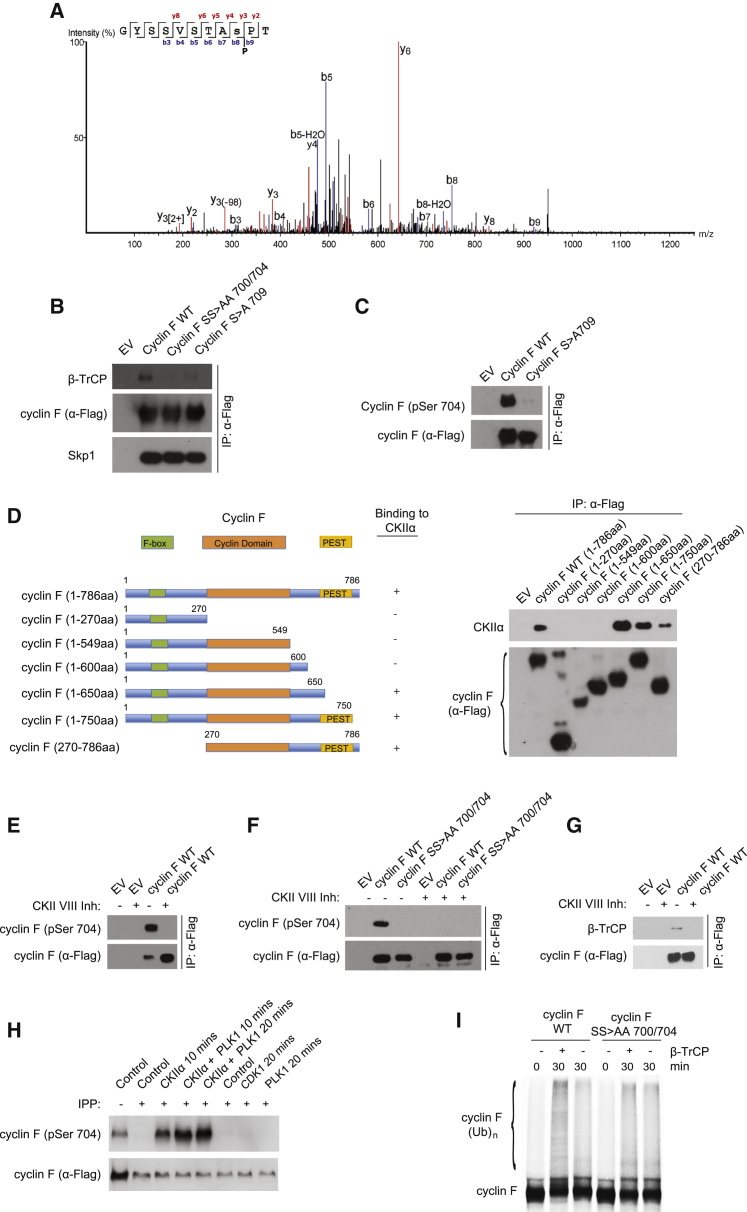
CKIIα Phosphorylates Cyclin F on S704 (A) Mass spectrometry-based mapping of S709 phosphorylation in cyclin F. The tandem mass spectrometry (MS/MS) spectrum of the peptides 701–711 from human cyclin F after elastase digestion. Fragment ions of the b and y series are indicated in blue and red, respectively. A complete list of fragment ions is in Figure S3D. (B and C) HEK293T cells transfected with cyclin F WT, SS>AA 700/704, S>A709 (B) or cyclin F WT and S>A709 (C) were immunoprecipitated (IP) with anti-FLAG resin and immunoblotted as indicated. (D) Left panel: schematic representation of cyclin F WT and truncated fragments, highlighting F-box, the cyclin domain, and PEST. Cyclin F mutants interacting with CKIIα are designated with the + symbol. Right panel: HEK293T cells transfected with empty vector (EV), FLAG-tagged cyclin F WT, or the indicated FLAG-tagged cyclin F truncated fragments were IP with anti-FLAG resin and immunoblotted as indicated. (E) HEK293T cells transfected with the indicated plasmids were treated with CKII inhibitor VIII for 24 hr, IP with anti-FLAG resin, and immunoblotted as indicated. (F) HeLa cells stably expressing an empty vector (EV), cyclin F WT, and SS > AA 700/704 treated with CKII inhibitor VIII for 24 hr were IP with anti-FLAG resin and immunoblotted as indicated. (G) HEK293T cells transfected with the indicated plasmids and treated with CKII VIII inhibitor were IP with anti-FLAG resin and immunoblotted as indicated. (H) FLAG-tagged cyclin F isolated from HEK293T was dephosphorylated by treatment with lambda phosphatase (λPPase±). After dephosphorylation, cyclin F was incubated with the indicate kinases for minutes and immunoblotted as indicated. IPP, incubation with λPPase. (I) Autoradiography of ^35^S-labeled *in vitro*-translated cyclin F (WT or S700A/S704A) used for an *in vitro* ubiquitylation assay and carried out in the presence or absence of unlabeled *in vitro*-translated β-TrCP1 (as indicated) and in the presence of purified recombinant CKIIα.

The preceding results show that β-TrCP controls cyclin F in a phosphorylation-dependent manner during mitosis. Therefore, we investigated the kinase or kinases responsible for these phosphorylation events. Considering the importance of PLK1 in mitosis (Bruinsma et al., 2012) and the proximity of the cyclin F degron to putative PLK1 recognition sites, we asked whether PLK1 could interact with and phosphorylate cyclin F. To this end, we tested the binding of cyclin F WT and cyclin F truncated mutants to PLK1. PLK1 interacted with cyclin F WT and two truncation mutants (1–750 and 270–786) (Figure S3A). To understand whether this residue is phosphorylated *in vivo*, we analyzed by liquid chromatography tandem mass spectrometry (MS) cyclin F purified from HEK293T cells to identify its phosphorylation sites. Because an arginine-rich region of cyclin F was not covered upon digestion with trypsin, we digested cyclin F with elastase and chymotrypsin and performed MS/MS to detect phosphorylation sites. Using this approach, we obtained a comprehensive map of cyclin F phosphorylation and secondary modifications (Figure S3B). We found that cyclin F phosphorylation is enriched at the C terminus, where the regulatory modifications of cyclin F cluster. Furthermore, MS/MS detected phosphorylation of cyclin F at both S704 and S709 in cells (Figure 3A; Figures 3C and S3D). Moreover, we found that the lack of S709 prevented binding to β-TrCP (Figure 3B). S709 is not within the minimal requirement for β-TrCP interaction; thus, we reasoned that the S709 phosphorylation could somehow affect the phosphorylation within the TSGXXS degron. Mutation of cyclin F in S709 abolished the phosphorylation at S704 (Figure 3C), indicating that S709 residue phosphorylation is a prerequisite for S704 phosphorylation in cells. We speculated that PLK1 could interact with S709 to mediate phosphorylation of S704; however, after treating cells with BI2536, a specific PLK1 inhibitor, we did not detect changes in the phosphorylation of S700 or S704, indicating that PLK1 does not participate in the regulation of cyclin F mediated by β-TrCP (Figure S3E).

To identify other potential kinases mediating S700 and S704 phosphorylation, we performed liquid chromatography (LC)-MS analysis of cyclin F interacting partners and identified known components of the SCF complex, known substrates (i.e., RRM2, CDC6, and Exo1), as well as CKIIα (Table S1). We tested the binding of cyclin F WT and cyclin F truncated mutants to endogenous CKIIα. CKIIα bound a C-terminal portion of cyclin F, encompassing aa 600 to 650 (Figure 3D). To understand whether CKIIα inhibition affected cyclin F phosphorylation, we expressed cyclin F WT in HEK293T cells and treated them with CKIIi VIII, a small molecule inhibitor of CKIIα. Our results revealed that phosphorylation of cyclin F at S704 was abolished upon CKIIα inhibition (Figure 3E), while cyclin F phosphorylation at S700 was impaired, but not abolished (Figure S3F). Similar results were obtained in HeLa cells stably expressing low levels of cyclin F (Figure 3F; Figure S3G). Results from three independent experiments were quantified (Figure S3H). The binding between cyclin F and β-TrCP was prevented upon CKIIα inhibition (Figure 3G).

The preceding data suggested that CKIIα phosphorylates cyclin F on S704. To further prove that CKIIα can phosphorylate the S704, we tested the ability of CDK1, PLK1, and CKIIα to phosphorylate S704 *in vitro*. The addition of CKIIα to purified cyclin F was sufficient to induce phosphorylation of S704. In contrast, PLK1 and CDK1 were unable to phosphorylate S704 of cyclin F (Figure 3H).

To understand whether the stability of cyclin F is controlled by CKIIα, we measured the cyclin F half-life after inhibition of CKIIα. We observed that the half-life of cyclin F is increased upon inhibition of CKIIα (Figure S4A).

To further establish the mechanism of ubiquitylation of cyclin F by β-TrCP and dependency on phosphorylation of S700 and S704, we reconstituted the ubiquitylation *in vitro* using reticulocyte lysates as previously done (Raducu et al., 2016). Ubiquitylation of cyclin F was promoted by the addition of β-TrCP in the reaction, while the presence of the S700 and S704 mutation impaired β-TrCP-mediated ubiquitylation (Figure 3I). Residual ubiquitylation observed in the reaction is likely due to cyclin F autoubiquitylation activity.

### β-TrCP-Mediated Degradation of Cyclin F Promotes Mitotic Progression

**Figure 4:**
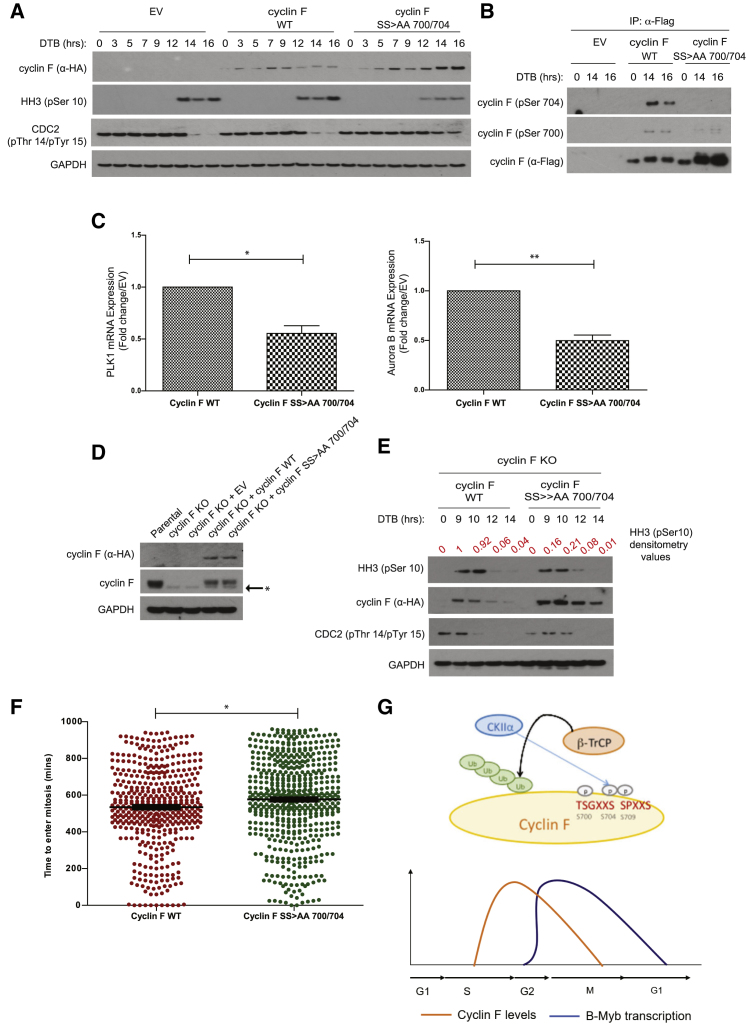
β-TrCP-Mediated Degradation of Cyclin F Promotes Mitotic Progression (A) HeLa cells stably expressing an empty vector (EV), cyclin F WT, and SS > AA 700/704 were synchronized by a DTB. Cells were collected at the indicated hours after release, lysed, and immunoblotted as indicated. (B) HeLa cells stably expressing an empty vector (EV), cyclin F WT, and SS > AA 700/704 were synchronized as in (A). Extracts were collected at the indicated hours, immunoprecipitated (IP) with anti-FLAG resin, and immunoblotted as indicated. (C) Quantitative real-time PCR analysis of PLK1 (left panel) and Aurora B (right panel) mRNA levels in HeLa cells stably expressing the indicated plasmids. Student’s t test. ^∗^p < 0.05, ^∗∗^p < 0.01. (D) HeLa cyclin F knockout (KO) cells infected with retroviruses expressing the indicated plasmids were immunoblotted as indicated. The asterisk corresponds to an unspecific band detected by the cyclin F antibody. (E) HeLa cyclin F knockout (KO) cells stably expressing cyclin F WT and SS > AA 700/704 were synchronized as in (A). Cells were collected at the indicated hours, lysed, and immunoblotted as indicated. (F) HeLa cyclin F knockout (KO) cells stably expressing cyclin F WT and SS > AA 700/704 were synchronized as in (A). Time-lapse microscopy was performed for 16 hr after release and analyzed. Student’s t test. ^∗^p < 0.05. (G) Scheme outlining the mechanism of cyclin F recognition by β-TrCP and its relevance.

The findings presented earlier demonstrate that β-TrCP regulates cyclin F during mitosis. To understand whether defective control of cyclin F impairs cell-cycle progression, we synchronized, by DTB, HeLa cells stably expressing an empty vector (EV) control, cyclin F WT, or the cyclin F SS > AA 700/704 mutant. The levels of cyclin F WT oscillated through the cell cycle, with a peak in G2 (7–9 hr after DTB release) (Figure 4A), as previously reported for endogenous cyclin F (D’Angiolella et al., 2010, D’Angiolella et al., 2012). This shows that the expression of cyclin F from an exogenous promoter does not affect its cell-cycle regulation. We also found that the cyclin F WT was phosphorylated when cells entered mitosis and that the cyclin F SS > AA 700/704 mutant was stable during mitosis (Figures 4A and 4B). The stabilization of cyclin F due to the mutation of S700 and S704 (Figure 4B) induced a reduction of histone H3 phosphorylation on serine 10 and prolonged CDC2 phosphorylation on threonine 14 and tyrosine 15 (Figure 4A), both markers of a delayed mitotic progression.

It has been previously shown that cyclin F controls the activation of B-Myb target genes by counteracting the cyclin A2-mediated phosphorylation of B-Myb (Klein et al., 2015). Thus, an accumulation of cyclin F in mitosis could impair mitosis progression by blocking the transcription of B-Myb target genes, such as PLK1 and Aurora B. To test the latter hypothesis, we analyzed the mRNA levels of PLK1 and Aurora B. The results of this analysis showed that, compared to cells expressing cyclin F WT, the mRNA levels of both PLK1 and Aurora B were significantly reduced in cells expressing SS > AA 700/704 (Figure 4C). The effect of SS > AA 700/704 on the mRNA levels of PLK1 and Aurora B levels were reverted by expressing B-Myb (Figure S4B).

The delayed mitotic progression and defective transcriptional control of PLK1 and Aurora B could simply be due to increased expression of cyclin F from an exogenous promoter. To exclude this possibility, we generated cyclin F knockout HeLa cells using CRISPR/Cas9 technology by a single-guide RNA. Of several transfected cells, we identified one clone (no. 28) to be a cyclin F knockout clone (Figure S4C). In the clone knockout for cyclin F, we re-introduced cyclin F WT or the cyclin F SS > AA 700/704 mutant. The expression of cyclin F from an exogenous promoter was lower than the expression of endogenous cyclin F in parental HeLa cells. However, the expression of cyclin F WT was identical to that of cyclin F SS > AA 700/704 (Figure 4D), we performed our experiments comparing these two cell lines. Similar to our previous findings, expression of cyclin F SS > AA 700/704 induced a reduction of histone H3 phosphorylation at serine 10 and a prolonged phosphorylation of CDC2 on threonine 14 and tyrosine 15 (Figure 4E). We also assessed the ability of these stable cell lines to exit mitosis after prolonged mitotic block with nocodazole. Cyclin F S700- and S704-expressing cells presented a reduction of histone H3 phosphorylation on serine 10. Furthermore, these cells showed a delay in mitosis exit due to prolonged phosphorylation of histone H3 on serine 10 and delayed phosphorylation of CDC2 at threonine 14 and tyrosine 15 (Figure S4D). Lastly, we synchronized cells expressing either cyclin F WT or the cyclin F SS > AA 700/704 mutant and monitored the time necessary for the cells to complete mitosis by time-lapse microscopy. We found that cells expressing cyclin F SS > AA 700/704 had a significant delay in mitotic progression compared to cells expressing cyclin F WT (Figure 4F).

Altogether, these findings indicate that the degradation of cyclin F by β-TrCP favors a mitotic B-Myb transcriptional program for mitotic progression.

## Discussion

In this study, we show that cyclin F is recognized by β-TrCP through a TSGXXS motif after phosphorylation of two serine residues (S700 and S704). We provide evidence that the phosphorylation of S704 is regulated by S709 and that the kinase responsible for the phosphorylation of S704 is CKIIα.

We have not identified the kinase or kinases responsible for phosphorylation of residues S709 and S700, although we show by LC-MS that these sites are phosphorylated *in vivo*. It is possible to predict that the kinases phosphorylating S709 and S700 are CDK1/2 and Aurora B, respectively. The phosphorylation of cyclin F by these kinases would ensure rapid degradation of cyclin F to amplify the signaling cascade required for the biological events occurring in mitosis.

It has been suggested that cyclin F is phosphorylated by CKIIα at S621 and S709 to control its activity (Lee et al., 2017). However, the biochemical mechanism of this regulation is unclear, because the S621 residue is far from the F-box motif required for the ubiquitylation activity of cyclin F. We observe that CKIIα interacts with the C-terminal region of cyclin F, which includes the S621 residue. Our data indicate that CKIIα promotes the recognition of cyclin F by β-TrCP, thus regulating its stability and abundance.

Similar to other cyclins, cyclin F has a relevant role in regulating proper cell-cycle progression. Fine-tuning of cyclin F levels through β-TrCP is necessary for cells’ timely control of mitotic progression. Specifically, our data are in accordance with a role of cyclin F in controlling B-Myb activity and, consequently, transcription of genes necessary for a proper mitosis. Given the crucial role of B-Myb in cellular senescence, it is tempting to speculate that the regulation of cyclin F by β-TrCP might be also involved in regulating cellular senescence. In addition, evidence suggests that cyclin F activity is controlled by AKT (Choudhury et al., 2017). AKT controls the transition from the G2 to the M phase of the cell cycle, as well as senescence (Zoncu et al., 2011). In this scenario, metabolic and environmental cues could converge on cyclin F to modulate mitotic progression.

It has been shown that cyclin F controls the degradation of the CDH1 subunit of the APC/C and, vice versa, that APC/C^CDH1^ mediates the degradation of cyclin F (Choudhury et al., 2016). It is possible that low levels of cyclin F, because of its β-TrCP-mediated degradation in mitosis, guarantee high CDH1 levels and activity at mitosis exit. We observe that expressing a mutant of cyclin F affects the progression through mitosis. Cyclin F has multiple substrates; thus, it is difficult to ascribe the phenotype observed to a single degradation event.

The effect of expressing a non-degradable mutant of cyclin F leads to a slight but reproducible delay in the mitosis progression. This is likely because cell-cycle control needs to be robust, with multiple redundant pathways controlling the same transition. Cyclin F fine-tunes cell-cycle transitions but is not a main driver. Our findings provide the basis to alter the levels of cyclin F in a physiological manner to study the multiple implications of altered cyclin F levels in controlling cell-cycle progression.

## Experimental Procedures

### Cell Culture

HEK293T, HeLa, and U2OS (ATCC, American Type Culture Collection) were grown in DMEM (Invitrogen). All cell lines were cultured in a humidified incubator at 37°C with 5% CO_2_ in the indicated culture medium containing 10% fetal bovine serum (Invitrogen), supplemented with 100 IU/mL penicillin (Corning) and 100 μg/mL streptomycin (Corning) solutions.

### Statistical Analysis

Quantitative analysis of band intensity was performed using ImageJ program. Data are expressed as means ± SEM. Statistical analyses were performed by using GraphPad Prism software. Differences between groups were analyzed by Student’s t test in Excel (paired, one-tailed distribution), and p < 0.05 was considered statistically significant.

### Antibody Generation

Two rabbit polyclonal antibodies against the following peptides of cyclin F were raised from 694 to ∼707: GKDVTTSpGYSSVST and GKDVTTSGYSSpVST. To ensure the specific recognition of phosphorylated cyclin F, the antibodies were affinity purified against a phosphorylated peptide and absorbed against a 694-GKDVTTSGYSSVST-707 non-phosphorylated peptide.

### Gene Silencing by siRNA

An siRNA oligonucleotide sequence targeting β-TrCP (5′-GUGGAAUUUGUGGAACAU-3′) (Dharmacon) and targeting both 515–535 of human β-TrCP1 and 262–282 of human β-TrCP2 was transfected in HeLa and U2OS cells using the HiPerfect (Thermo Fisher Scientific) transfection reagent according to the manufacturer’s instructions. A non-targeting siRNA (Dharmacon) served as a negative control.

## Data and Software Availability

The accession number for the mass spectrometry proteomics data reported in this paper is PRIDE: PXD010737, courtesy of the ProteomeXchange Consortium via the PRIDE partner repository.

## Supplemental Information

Document S1. Supplemental Experimental Procedures, Figures S1–S4, and Table S1

Document S2. Article plus Supplemental Information
