# Supplementary material for: β-TrCP- and Casein Kinase II-Mediated Degradation of Cyclin F Controls Timely Mitotic Progression
Source: Cell Rep. 2018 Sep 25;24(13):3404–12. doi: 10.1016/j.celrep.2018.08.076 (PMC6172692; doi:10.1016/j.celrep.2018.08.076)
Supplement: Document S1. Supplemental Experimental Procedures, Figures S1–S4, and Table S1 [file mmc1.pdf]

Cell Reports, Volume *24*

Supplemental Information

β-TrCP- and Casein Kinase II-Mediated Degradation of Cyclin F Controls Timely Mitotic Progression

Ioanna Mavrommati, Roberta Faedda, Giovanni Galasso, Jie Li, Kamila Burdova, Roman Fischer, Benedikt M. Kessler, Zunamys I. Carrero, Daniele Guardavaccaro, Michele Pagano, and Vincenzo D'Angiolella

SUPPLEMENTARY EXPERIMENTAL PROCEDURES

Cell cycle synchronisation

For G1/S synchronisation, HeLa cells were cultured in the presence of 2.5mM thymidine (Sigma) for 16 hours, washed twice with Phosphate Buffer Saline (PBS), once with medium and cultured in fresh medium for 8 hours. 2.5mM thymidine was then added to cells for further 16 hours, washed twice with PBS, once with medium and cultured into fresh medium. siRNA oligos were transfected between the first and second thymidine block. To trap cells in prometaphase, 100ng/ml nocodazole (MP Biomedicals) was added seven hours after the second release from thymidine block.

To enrich the G2 and M cell populations, cells were cultured in 2.5mM thymidine for 16 hours, washed twice with PBS, once with medium and cultured in fresh medium for 4 hours. Cells were then cultured in the presence of nocodazole for 16 hours.

Chemical Inhibitors

The following inhibitors were used: Casein Kinase II Inhibitor VIII (Merck) at 2.5μM, MLN4924 (Cell signalling) at 2μM, MG132 (Sigma) at 10μM and cycloheximide (CHX) (Sigma Aldrich) at 50μg/ml.

Plasmids generation

Cyclin F and cyclin F fragments plasmids have been previously described (D'Angiolella et al., 2010). Cyclin F mutants where generated by site directed mutagenesis on the pcDNA3 Flag-cyclin F backbone. Cyclin F WT and mutants were isolated from pcDNA3 and inserted into pBabe by sub-cloning. Flag-tagged β-TrCP1 (Fbxw1), β-TrCP2 (Fbxw11), Fbxw2, Fbxw5, Fbxw7, Cdh1, Cdc20 and Myc-Ubiquitin were described before (D'Angiolella et al., 2010).

Transient plasmid expression

For transient plasmid transfection, HEK293T cells were seeded the previous day prior to transfection using the reagent "PEI Max", Polyethylenimine HCl Max (Polysciences).

Stable cell lines generation

For retrovirus production, a pBabe-puro (Addgene, 1764) retroviral vector containing the gene of interest was co-transfected into HEK293T with the pUMVC Gag/Pol vector (Addgene, 8449) packaging vector and pCMV-VSV-G (Addgene, 8454) envelope-encoding vector. For transduction, cells were seeded at 30% confluency. 24 hours later, fresh medium was added along with the virus-containing media at a 1:1 ratio, in the presence of 8μg/mL polybrene, and incubated for 16 hours. This was followed by 2μg/mL puromycin selection for 72 hours. Expression of the protein of interest was confirmed by Western blot.

For CRISPR cyclin F KnockOut (KO) cell lines, sgCCNF (5'-CTCAAGATGGTCAGGTTTCG-3') was cloned into pSpCas9 (BB) pX330 vector (Addgene plasmid ID 42230) as previously described (Bauer et al., 2015). Single cell clones were sorted and cyclin F loss was identified by Western blot.

Antibodies

Cyclin F: 1:4000, SantaCruz Biotechnology, Cat. No. sc-95)

Cyclin F (Ser700): custom generated by Yenzyme

Cyclin F (Ser704): custom generated by Yenzyme

FLAG: 1:1000, Sigma-Aldrich, Cat. No. F7425

GAPDH: 1:5000, Thermo Fisher Scientific, Cat. No. MA5-15738

HA (16B12): 1:1000, Biolegend, Cat. No. 901501

pCDC2 (Thr14/Tyr15): 1:1000, Santa Cruz Biotechnology, Cat. No. sc-12340-R

pHH3 (Ser10): 1:1000, Millipore, Cat. No. 6570

RRM2 (N-18): 1:1000, Santa Cruz Biotechnology, Cat. No. sc-10844

SKP1: 1:1000, Cell Signalling, Cat.No. 2156

β-TrCP: 1:1000, Cell Signalling, Cat.No 4394

Rabbit IgG: Santa Cruz Biotechnology, Cat. No sc-2027

Aurora A pT288, Cell Signalling Cat.No. 3875

CKIIα substrate, Cell Signalling Cat.No. 12170

mRNA quantification

Total RNA was extracted from cells by using RNease Mini Kit (QIAGEN), according to manufacturer's instructions. 100ng of total RNA was retro-transcribed by using Superscript III kit (Invitrogen), according to manufacturer's instructions. The complimentary DNA (cDNA) obtained, was used as templates for qPCR. RT-qPCR was performed using 2X Maxima SYBR green/Fluorescein qPCR mix (Thermo Fisher). GAPDH cDNA was also amplified as an internal control. The relative changes in gene expression quantification were calculated using the relative ddCT analysis mode of the ABI 7500 Fast Real-Time PCR system software. All experiments were performed in triplicate.

CCNF_FW: 5'- CCCCGAAGATGTGCTCTTTCA-3'

CCNF_Rev: 5'- GCCTTCATTGTAGAGGTAGGCT-3'

Plk1_FW: 5'- TGTTCGCGGGCAAGATTGT-3'

Plk1_Rev: 5'- GGCTGCGGTGAATGGATATTTC-3'

AuroraB_FW: 5'- CAGAGAGATCGAAATCCAGGC-3'

AuroraB _Rev: 5'- CTGTTCGCTGCTCGTCAAAT-3'

GAPDH_FW: 5'-ATGCCTCCTGCACCACCAAC-3'

GAPDH_Rev: 5'-GGGGCCATCCACAGTCTTCT-3'

Immunoprecipitation and Immunoblotting

HEK293T were transiently transfected with indicated plasmids with polyethylenimine. Cell lysis was performed with lysis buffer (50mM Tris pH 7.5, 150mM NaCl, 1mM EDTA, 5mM MgCl2, 10% glycerol and 0.1% NP-40) supplemented with phosphatase and protease inhibitors for at least 10 minutes on ice. Cell lysates were then immunoprecipitated with either anti-FLAG (Sigma Aldrich) or anti-HA (Biolegend) antibody conjugated to agarose resin. For immunoprecipitation of endogenous proteins, HEK293T cells were collected and lysed with lysis buffer. Cyclin F was immunoprecipitated with Protein G Sephasrose 4 Fast Flow (GE Healthcare). Rabbit IgG was used as a negative control. Elution of imuunoprecipitate was carried out with NuPAGE® LDS sample buffer (Thermo Fisher Scientific) supplemented with β-mercaptoethanol (Sigma-Aldrich) followed by incubation at 95°C for 5 minutes. Samples were loaded on 7-14% Tris-glycine gels (Invitrogen), run at 120V for 90 minutes with MES-buffer (Invitrogen), and transferred to PVDF membranes (Merck Millipore). Membranes were then incubated in PBS supplemented with 0.1% Tween 20 (PBST-T) (v/v) and 5% skimmed milk powder for 30-60 mins at room temperature, followed by overnight incubation with primary antibodies at +4°C or 1 hour incubation at room temperature. After washing with PBS-T, membranes were incubated with secondary antibodies for 1 hour at room temperature, washed with PBS-T and exposed to ECL (Thermo Scientific).

Ubiquitylation assay

EV, Flag-tagged Cyclin F or HA-tagged β-TrCP2 were co-transfected with 1μg Myc-tagged ubiquitin. At twenty four hours post transfection, cells were incubated with MG132 for four hours prior to lysis. Anti-Flag M2 agarose beads were used to immunoprecipitate cyclin F. The beads were washed four times in lysis buffer.

Samples were then incubated at 95°C for 10 minutes, resolved by SDS-PAGE and analysed by immunoblotting.

***In vitro* ubiquitylation assay**

In vitro ubiquitylation assay was previously described (Dorrello et al., 2006). Briefly, cyclin F ubiquitylation was performed in a volume of 10 μl containing 50 mM Tris pH 7.6, 5 mM $MgCl_2$, 0.6 mM DTT, 2 mM ATP, 2 μl *in vitro* transcribed/translated unlabeled β-TrCP1, 1.5 ng/μl E1 (Boston Biochem), 10 ng/μl Ubc3, 10 ng/μl Ubc5, 2.5 μg/μl ubiquitin (Sigma-Aldrich), 1 μM ubiquitin aldehyde (Sigma-Aldrich), 180 nM CK2α (Invitrogen), 1 μl ^{35}S-methionine-labelled *in vitro* transcribed/translated cyclin F as substrate. The reactions were incubated at 30°C for 30 minutes and analyzed by SDS-PAGE and autoradiography.

Time-lapse microscopy

HeLa cells were synchronised to G1/S phase by double thymidine block, released into fresh medium and transferred to Nikon Ti-E microscope with the Nikon Perfect Focus System (PFS). Imaging was undertaken with a 20x 0.75NA objective with the cells maintained in a constant atmosphere of 5% CO2 at 37°C throughout. Images were acquired as a 3x3 montage with a Hamamatsu Flash4 sCMOS detector at 5 minutes intervals for a total of 24 hours. Tracking of cells undergoing division was undertaken using the TrackMate plugin for Fiji. For detection a Difference of Gaussian (DoG) filter was utilised with a high threshold value to ensure only cells undergoing division were detected.

Tandem affinity purification and mass spectrometry

Analysis of immunoprecipitated endogenous cyclin F material was essentially performed as described previously (Adam et al., 2011). In brief, immunoprecipitated cyclin F was separated by SDS-PAGE, visualized by Coomassie staining, and gel

bands were excised and destained overnight in 50% methanol, 5% acetic acid in water. Proteins were reduced with 10 mM dithiothreitol and then alkylated using 20 mM iodoacetamide. Proteins were digested with 1 µg elastase or trypsin in 50 mM ammonium bicarbonate overnight at 37 °C, and resulting peptides were extracted from the gel with 50% acetonitrile, 5% acetic acid in water. Peptide material was analysed on an Acquity nano UPLC system (Waters) equipped with a 25 cm C18 column, 1.7 µm particle size (Waters) online coupled to an LTQ Orbitrap Velos (Thermo Scientific) as described (Adam et al., 2011). Raw MS data was processed and analysed using PEAKS software as described previously (Davis et al., 2017). The mass spectrometry proteomics data have been deposited to the ProteomeXchange Consortium via the PRIDE partner repository with the dataset identifier PXD010737 and 10.6019/PXD010737. Database: http://www.ebi.ac.uk/pride

Peptide cross-linking

The following peptides: 694-GKDVTTSGYSSVST-707 (nophos), 694-GKDVTTpSpGYSSpVST-707 (phos), 694-GKDVTTSpGYSSpVST-707 (Δ699), 694-GKDVTTpSGYSSpVST-707 (Δ700), 694-GKDVTTpSpGYSSVST-707 (Δ704) were conjugated to CNBr-activated Sepharose-4B according to the manufacturer's instruction.

***In vitro* kinase assay**

HEK293T cells were transfected with Flag-cyclin F WT for twenty four hours followed by immunoprecipitation with ANTI-FLAG® M2 Affinity Gel (Sigma-Aldrich). Immunopurified Flag-cyclin F was first de-phosphorylated by treatment with lambda phosphatase (New England Biolabs) and then incubated at 30°C for 25 minutes with 0.2 mM ATP and the indicated kinases in a 10 µl reaction of kinase buffer (25 mM Tris pH 7.5, 10 mM MgCl2, 2 mM DTT, 5 mM β-glycerophosphate,

0.1 mM sodium orthovanadate). Reaction products were stopped with Laemmli Sample buffer and then subjected to immunoblotting. For sequential *in vitro* kinase assay, immunopurified Flag-cyclin F was subjected to a first phosphorylation reaction (25 mins) with the indicated purified kinases. Samples were then washed three times in lysis buffer (50 mM Tris-HCl pH 7.5, 250 mM NaCl, 0.1% Triton X-100, 1 mM EGTA) to remove the first kinase and twice in kinase buffer. Samples were then subjected to a second phosphorylation reaction as described above.

REFERENCES

Adam, J., Hatipoglu, E., O'Flaherty, L., Ternette, N., Sahgal, N., Lockstone, H., Baban, D., Nye, E., Stamp, G.W., Wolhuter, K., *et al.* (2011). Renal cyst formation in Fh1-deficient mice is independent of the Hif/Phd pathway: roles for fumarate in KEAP1 succination and Nrf2 signaling. Cancer Cell *20*, 524-537.

Bauer, D.E., Canver, M.C., and Orkin, S.H. (2015). Generation of genomic deletions in mammalian cell lines via CRISPR/Cas9. J Vis Exp, e52118.

D'Angiolella, V., Donato, V., Vijayakumar, S., Saraf, A., Florens, L., Washburn, M.P., Dynlacht, B., and Pagano, M. (2010). SCF(Cyclin F) controls centrosome homeostasis and mitotic fidelity through CP110 degradation. Nature *466*, 138-142.

Davis, S., Charles, P.D., He, L., Mowlds, P., Kessler, B.M., and Fischer, R. (2017). Expanding Proteome Coverage with CHarge Ordered Parallel Ion aNalysis (CHOPIN) Combined with Broad Specificity Proteolysis. J Proteome Res *16*, 1288-1299.

Figure S1

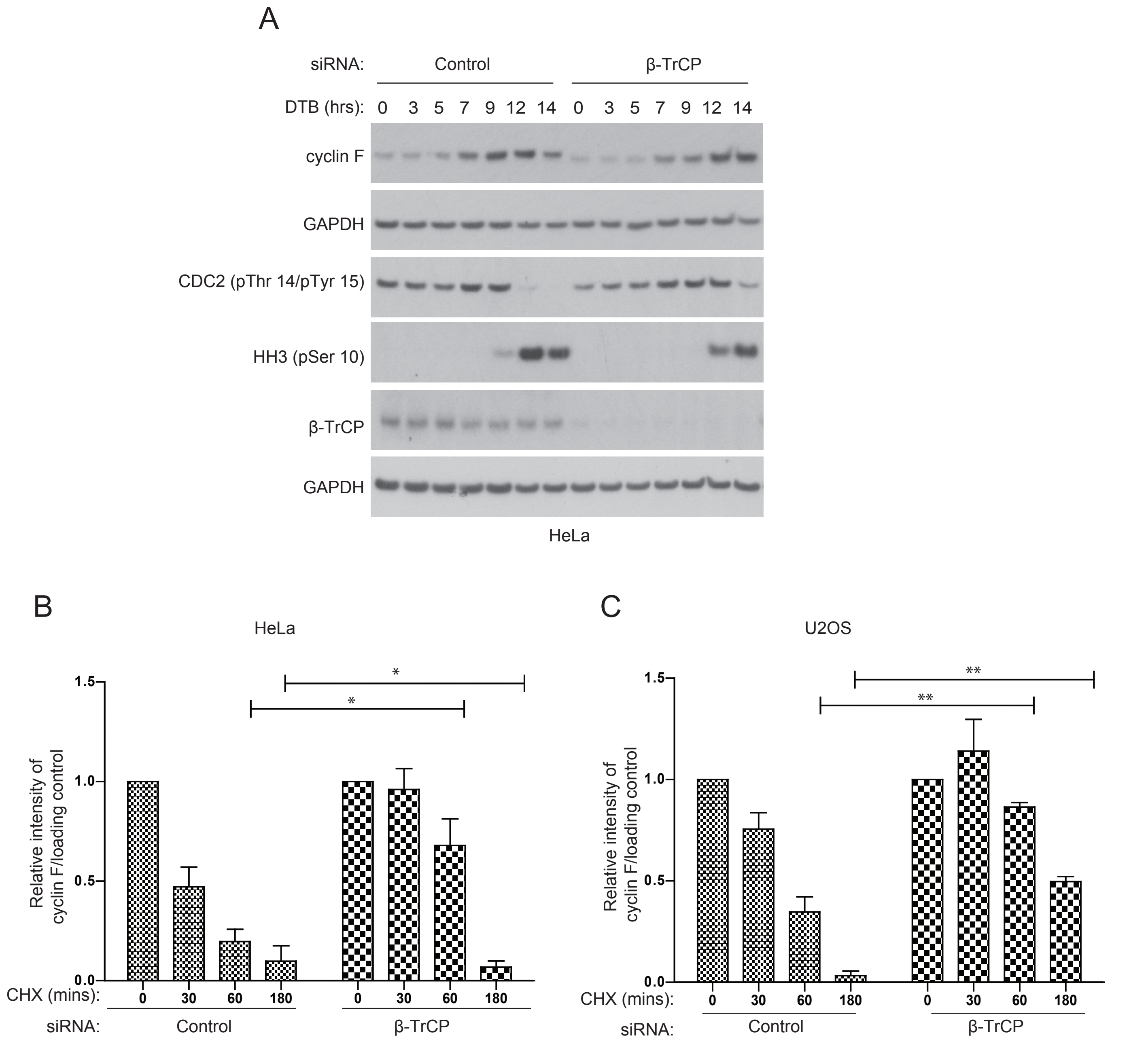

Figure S1: Cyclin F levels are controlled by β-TrCP. Related to Figure 1.
(A) HeLa cells transfected with indicated siRNA were synchronised by a DTB and released in fresh medium. Cells were collected at the indicated hours (hrs) after release, lysed and immunoblotted as indicated. (B) Western blot densitometry analysis of cyclin F half-life from HeLa cells experiments. Results are presented as a fold change over cyclin F protein level at time point zero for each siRNA and are means ±SEM of three independent experiments. Student t-test * $p<0.05$. (C) Western blot densitometry analysis of cyclin F half-life from U2OS cells experiments. Results are presented as a fold change over cyclin F protein level at time point zero for each siRNA and are means ±SEM of three independent experiments. Student t-test ** $p<0.01$.

Figure S2

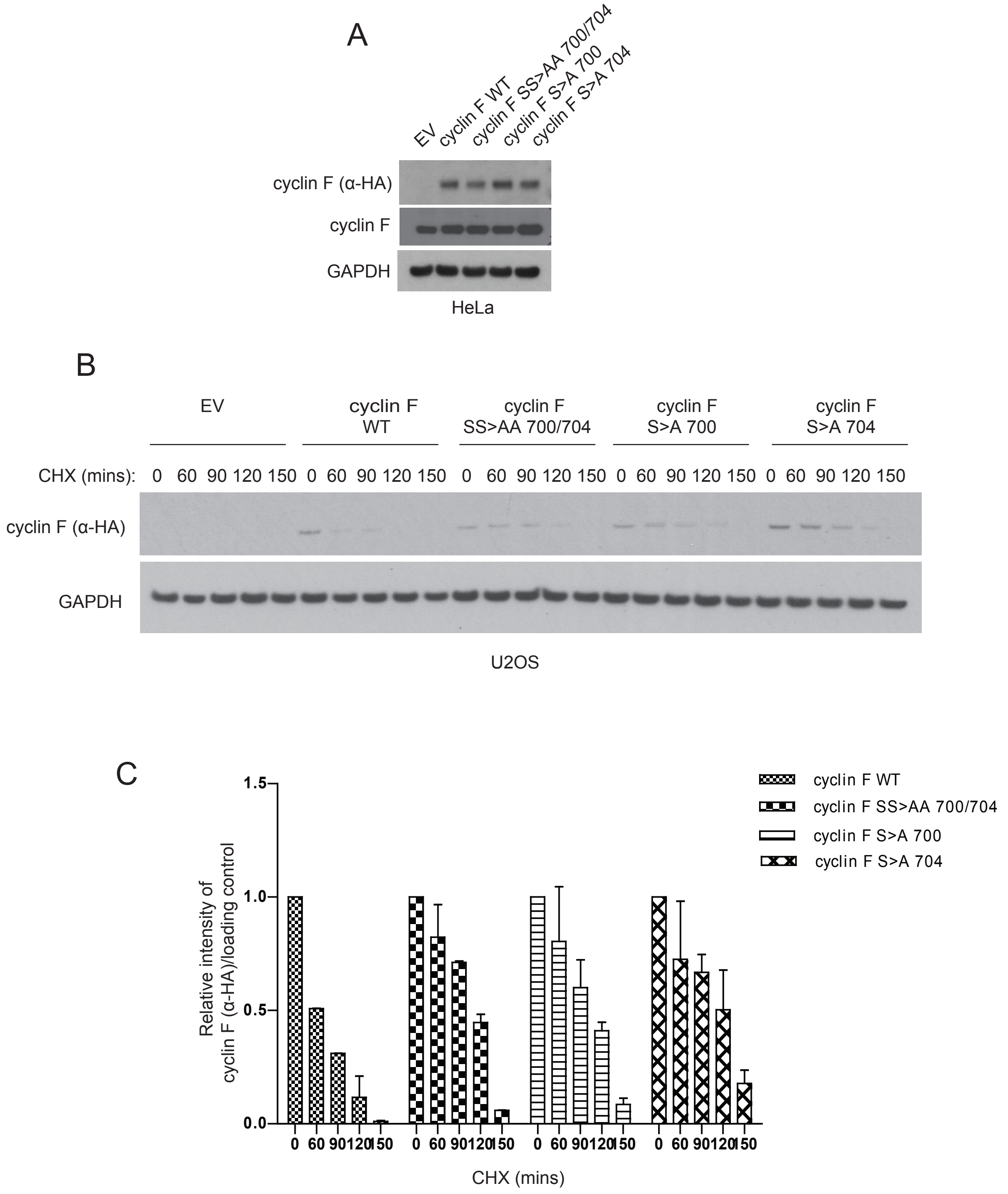

Figure S2: Cyclin F mutated in the β-TrCP 'degron' has a prolonged half-life. Related to Figure 2.
(A) HeLa cells stably expressing Empty Vector (EV), cyclin F WT and S>A 700, S>A 704 and SS>AA 700/704 mutants were collected, lysed and immunoblotted as indicated. GAPDH was used as a loading control. (B) U2OS cells stably expressing Empty Vector (EV), cyclin F WT and S>A 700, S>A 704 and SS>AA 700/704 mutants were treated with cycloheximide (CHX) for indicated minutes (mins). Cells were collected, lysed and immunoblotted as indicated. Representative image of two independent experiments is shown. (C) Western blot densitometry analysis of cyclin F. Results are presented as a fold change over cyclin F protein level at time point zero for each cell line and are means ±SEM of two independent experiments.

Figure S3

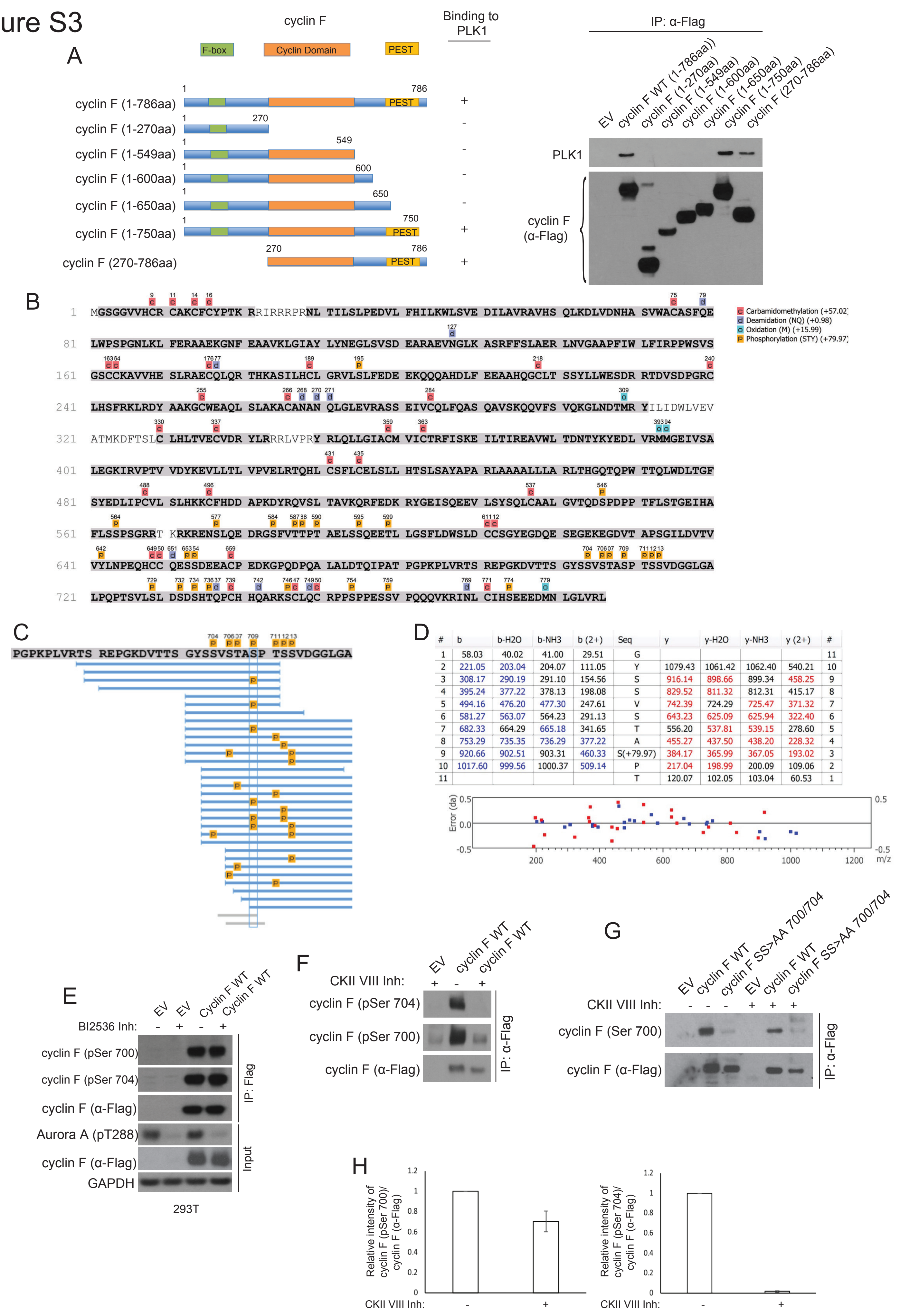

#	b	b-H2O	b-NH3	b (2+)	Seq	y	y-H2O	y-NH3	y (2+)	#
1	58.03	40.02	41.00	29.51	G					11
2	221.05	203.04	204.07	111.05	Y	1079.43	1061.42	1062.40	540.21	10
3	308.17	290.19	291.10	154.56	S	916.14	898.66	899.34	458.25	9
4	395.24	377.22	378.13	198.08	S	829.52	811.32	812.31	415.17	8
5	494.16	476.20	477.30	247.61	V	742.39	724.29	725.47	371.32	7
6	581.27	563.07	564.23	291.13	S	643.23	625.09	625.94	322.40	6
7	682.33	664.29	665.18	341.65	T	556.20	537.81	539.15	278.60	5
8	753.29	735.35	736.29	377.22	A	455.27	437.50	438.20	228.32	4
9	920.66	902.51	903.31	460.33	S(+79.97)	384.17	365.99	367.05	193.02	3
10	1017.60	999.56	1000.37	509.14	P	217.04	198.99	200.09	109.06	2
11					T	120.07	102.05	103.04	60.53	1

Figure S3: Map of cyclin F secondary modifications and phosphorylation by CKII Related to Figure 3.
(A) Left panel. Schematic representation of cyclin F WT and truncated fragments highlighting cyclin F fragments and domains: F-box, cyclin domain, PEST. Cyclin F mutants that interacted with endogenous PLK1 are designated with the (+) symbol. Right panel. HEK293T cells were transfected with empty vector (EV), Flag-tagged cyclin F wild-type (WT), or the indicated Flag-tagged cyclin F truncated fragments. Whole cell extracts were immunoprecipitated (IP) with anti-Flag resin, and immunoblotted as indicated. (B) Post Translational Modification landscape of endogenous cyclin F profiled by mass spectrometry. PEAKS analysis reveals sites of cysteine © carbamidomethylation (orange), asparagine/glutamine (N/Q) deamidation (blue), methionine (M) oxidation (light blue) and serine/threonine/tyrosine (S/T/Y) phosphorylation (yellow). (C) Hotspot of phosphorylation in the region of the TSGXXS motif. Serine (S) phosphorylation sites are indicated in yellow, and each blue line indicates a separate peptide fragment identified by mass spectrometry. (D) Table of fragmentation ions for the cyclin F derived peptide 701-711. Fragment ions of the b- and y- series that are detected by mass spectrometry are indicated in blue and red, respectively. (E) HEK293T cells were transfected with the indicated plasmids and treated with PLK1 inhibitor (BI2536) for twenty-four hours prior collection. Whole cells extracts were immunoprecipitated (IP) with anti-Flag resin and immunoblotted as indicated. (F) HEK293T cells were transfected with the indicated plasmids and treated with CKII inhibitor VIII for two hours prior collection. Whole cells extracts were immunoprecipitated (IP) with anti-Flag resin and immunoblotted as indicated. (G) HeLa cells stably expressing Empty Vector (EV), cyclin F WT and SS>AA 700/704 mutants were treated with CKII inhibitor VIII for twenty-four hours prior collection. Whole cell extracts were immunoprecipitated (IP) with anti-Flag resin, and immunoblotted as indicated. (H) Western blot densitometry analysis of S700 and S704 phosphorylation after treatment with CKIIα inhibitor VII. Results are presented as a fold change of S700 and S704 signal over cyclin F protein level and are means ±SEM of three independent experiments. Student t-test ** $p<0.01$.

Figure S4

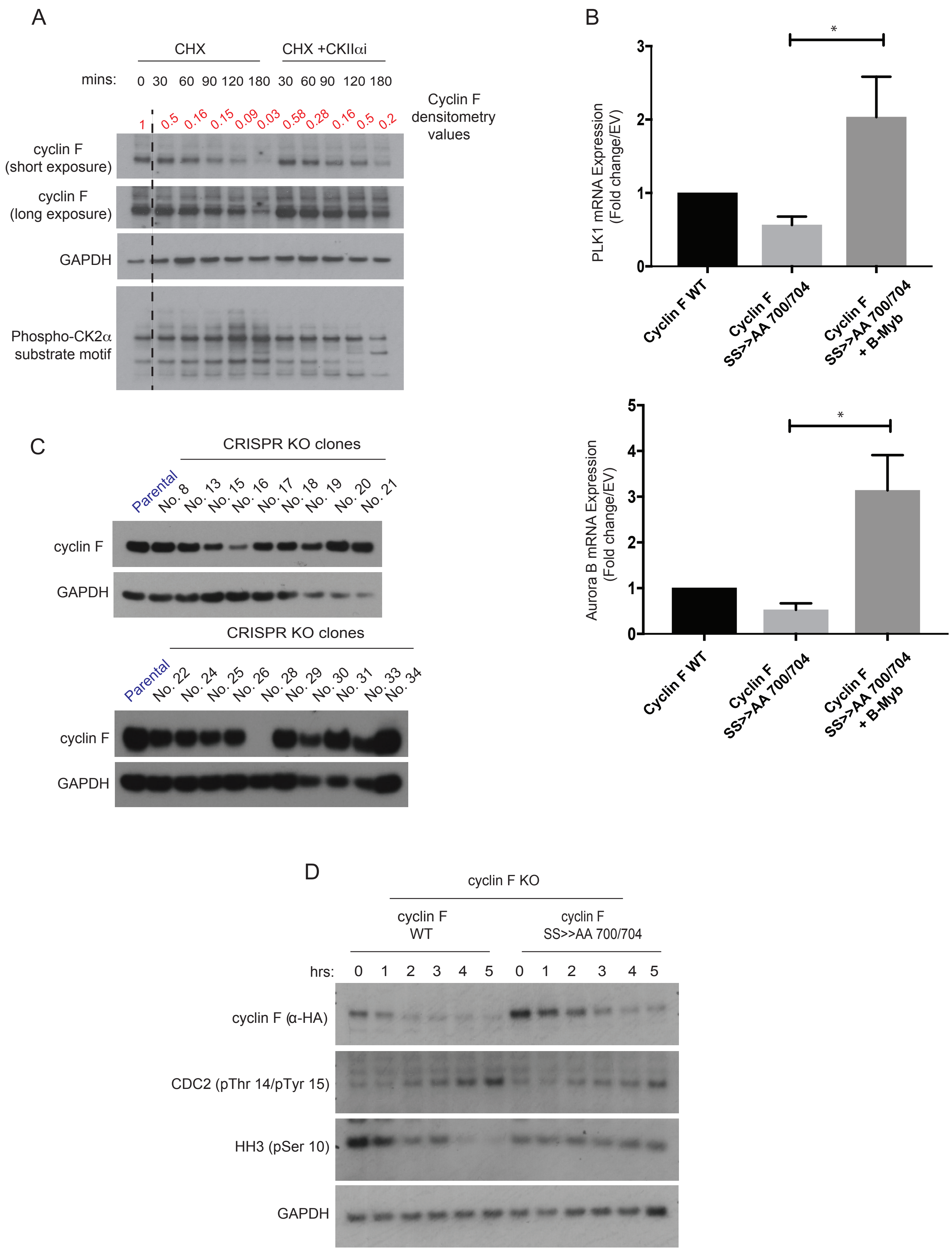

Figure S4: Cyclin F controls timely mitotic progression. Related to Figure 3 and 4.
(A) HeLa cells where synchronised in mitosis using nocodazole and treated with cycloheximide (CHX) for indicated minutes (mins). Cells were collected, lysed and immunoblotted as indicated. (B) Quantitative real-time PCR analysis of PLK1 (top panel) and Aurora B (bottom panel) mRNA levels in HeLa cells expressing the indicated plasmids. GAPDH was used as a house keeping gene. Student t-test * $p<0.05$, ** $p<0.01$.
(C) Western blot analysis of HeLa cyclin F KnockOut (KO) clones compared to parental cells. GAPDH was used a loading control.
(D) HeLa cyclin F KnockOut (KO) cells stably expressing cyclin F WT and SS>AA 700/704 were synchronised by nocodazole in mitosis and released in fresh medium for the indicated hours (hrs). GAPDH was used as a loading control.

Accession	Score	Mass	Matches	Pep(sig)	Sequences	Sequences (sig)	emPAI	Description
P41002	19884	89409	1040	683	65	59	47.88	Cyclin F
P63208	2061	18817	46	38	11	10	8.98	S-phase kinase associated protein 1
P68400	865	45229	57	40	19	18	6.73	Casein kinase II subunit alpha
P19784	700	41358	36	26	22	18	4.02	Casein kinase II subunit alpha'
Q13616	471	90306	33	18	20	16	0.83	Cullin-1
Q99741	384	63650	25	19	20	17	1.48	Cell division control protein 6 homolog
P67870	278	25268	21	14	10	7	2.05	Casein kinase II subunit beta
P31350	130	45134	10	5	8	5	0.42	Ribonucleoside-diphosphate reductase subunit M2
Q9UQ84	80	95185	3	3	3	3	0.11	Exonuclease 1

Supplementary Table1. Cyclin F interacting proteins identified by LC/MS analysis. Related to Figure 3. The table represents the summary of a Flag-tagged cyclin F immunoprecipitation experiment followed by LC/MS.
